# Commentary: Low expression of SOD and PRX4 as indicators of poor prognosis and systemic inflammation in colorectal cancer

**DOI:** 10.3389/fonc.2025.1739574

**Published:** 2025-12-17

**Authors:** Yan Zhang, Xiao Dong, Na Liu, Yonghong Li

**Affiliations:** 1 Gansu University of Chinese Medicine, Lanzhou, China; 2 Gansu Provincial Hospital, Lanzhou, China

**Keywords:** antioxidants, biomarkers, colorectal neoplasms, oxidative stress, prognosis

## 1 Introduction

As the global incidence and mortality rates of colorectal cancer (CRC) continue to rise, the identification of new biomarkers for predicting tumor prognosis and treatment response has become a central focus in cancer research (1). The role of the antioxidant enzyme system in tumorigenesis, progression, and chemoresistance is garnering increasing attention, particularly the antioxidants superoxide dismutase (SOD) and peroxiredoxin 4 (PRX4), which are increasingly recognized for their strong association with the clinical characteristics and prognosis of CRC (2, 3). The study by Sanghyun An et al. investigates low SOD and PRX4 expression as indicators of poor prognosis and systemic inflammation in CRC, while also exploring the relationship between these biomarkers and tumor aggressiveness (4). This commentary will critically assess the study’s methodological limitations, sample size, statistical robustness, and the generalizability of its conclusions, alongside discussing its potential clinical implications and suggesting improvements to further advance the field.

## 2 Commentary and discussion

The primary strength of this study lies in its innovation. The research establishes a novel link between the low expression of SOD and PRX4 and the malignant characteristics, systemic inflammation, and poor prognosis of colorectal cancer. It represents the first attempt to integrate these two antioxidant biomarkers with the clinical features of CRC, thereby providing a new theoretical foundation. This innovative approach offers potential biomarkers for personalized treatment strategies and supports clinical decision-making, particularly in predicting distant metastasis and inflammatory responses. Moreover, the study employs reliable biomarker detection techniques, such as enzyme-linked immunosorbent assay (ELISA), yielding high-quality data that can inform future research.

Although this study provides innovative insights, it has several limitations that affect the reliability and generalizability of its conclusions. While the association between low expression of these biomarkers and poor prognosis is interesting, the study’s small sample size (n=70) and lack of statistical significance in multivariable analysis raise concerns about the robustness of these conclusions. In particular, the study’s reliance on Cox regression without addressing overfitting or the impact of confounding factors limits the applicability of the findings. These issues should be addressed in future research to enhance the reliability of these biomarkers as clinical tools (5). The study by An et al. provides valuable insights into the potential role of low expression levels of SOD and PRX4 as biomarkers of poor prognosis in colorectal cancer. However, the conclusions drawn regarding their association with disease-free survival (DFS) and overall survival (OS) require careful consideration. Although the study identifies a trend between low SOD and PRX4 expression and poor prognosis, the multivariable analysis did not establish statistically significant independent prognostic roles for these biomarkers in DFS and OS. This finding highlights the need for further validation with larger sample sizes and stronger statistical power to support the clinical utility of these biomarkers. Thus, the conclusions drawn may be overly reliant on statistical chance and lack sufficient power to support widespread clinical application. To enhance the robustness of the findings, it is recommended that future studies conduct *a priori* power analyses and report detectable effect sizes for given sample sizes, while also considering the inclusion of large, multicenter cohorts (6). Additionally, employing regularization regression techniques, such as LASSO-Cox, could mitigate overfitting risks and enhance the stability of the analysis (7). Key conclusions should be validated through cross-validation or bootstrap stability analyses.

Another significant concern is the use of protein expression units in the figure, which are unclear and inconsistent with the existing literature. The study also categorizes antioxidant biomarker expression levels using the first quartile as a cutoff point to separate patients into high-expression and low-expression groups. However, this method may lack stability, especially given the small sample size, and varying cutoff points across different studies can reduce the comparability of results (8). The absence of sensitivity analyses for cutoff points prevents an evaluation of how the choice of cutoff impacts the study’s conclusions, potentially leading to overinterpretation of the findings. Future research should standardize the units used for protein expression and carefully justify the selection of cut-off points to ensure comparability with other studies. Additionally, sensitivity analyses should be conducted at multiple thresholds, such as the median, 75th percentile, or data-driven optimal split points, to assess their impact on key conclusions (9). Modeling biomarkers as continuous variables, such as inputting SOD and PRX4 levels directly into the Cox regression model, can help avoid biases introduced by cutoff points, allowing for a more robust evaluation of the biomarkers’ prognostic valu (10).

Moreover, this study measured antioxidant biomarker levels in tumor tissues only at the time of surgery, lacking dynamic data before and after treatment or during follow-up. This limitation restricts the ability to track changes in antioxidant biomarker levels associated with disease progression or treatment interventions, making it difficult to elucidate causal relationships between these biomarkers and colorectal cancer progression (11). Under the current design, the levels of antioxidant biomarkers may be influenced by various factors, such as treatment protocols and inflammatory states, complicating the distinction between correlation and causation. To enhance the capacity for causal inference in future research, prospective cohort studies should be implemented, with multiple time-point assessments of antioxidant biomarker levels taken before treatment, after treatment, and at recurrence, along with conducting time-dependent survival analyses (12). Additionally, integrating structural equation modeling or causal inference methods to explore the potential causal pathways of changes in antioxidant biomarkers during follow-up will provide clearer insights into the underlying biological mechanisms (13).

As for the multivariable analysis, the selection threshold for variables was set at p<0.20. While this approach is effective for preliminary screening, the lack of detailed descriptions regarding the stepwise regression process hampers understanding of the rationale behind variable selection (14). Furthermore, the study did not clarify how missing data were managed, such as whether multiple imputation strategies were utilized, nor did it report the proportion of missing data. Inadequate handling of missing data can introduce bias and adversely affect the accuracy and reliability of effect estimates. To enhance transparency and reproducibility, future studies should thoroughly outline the variable selection process along with the criteria for entering and exiting in stepwise regression, and adequately assess collinearity. Moreover, appropriate imputation methods for missing data, such as multiple imputation, should be implemented, with a comparison of results before and after imputation, alongside reporting the proportion and management strategies for any missing data.

Although multivariable Cox regression analysis was conducted, the study may still be influenced by uncontrolled confounding factors such as variations in treatment regimens, including surgical methods, chemotherapy, radiotherapy, and postoperative treatments, as well as molecular characteristics like KRAS, NRAS, and BRAF mutations and microsatellite instability status, along with inflammatory states. The presence of these confounding factors can obscure the true effects of antioxidant biomarkers or distort the strength of their associations (15). To better control for confounding bias, future research should systematically include these critical clinical and molecular features within their models and could employ propensity score matching analysis to balance inter-group differences. Additionally, conducting sensitivity analyses to assess the impact of excluding or including specific subgroups, such as those defined by treatment regimens or mutation types, on the results will further enhance causal interpretation.

While the study demonstrated that low-expression groups of SOD and PRX4 had poorer prognosis in terms of 5-year DFS and OS, the Cox regression analysis failed to confirm their independent prognostic value within the multivariable model. As a result, although the text presents a description of trend relationships that carries academic value, it has not achieved statistical significance and should clearly differentiate between statistical significance and potential biological significance or trend signals (16). Future research should exercise caution in the use of statistical significance terminology in their conclusions and discussions and clearly articulate interpretative methods when statistical significance is not reached to avoid overinterpretation. Furthermore, presenting the stability of effect sizes and confidence intervals in figures and supplementary analyses can assist readers in intuitively assessing the robustness of the findings. Furthermore, while the study focuses on the combined role of SOD and PRX4, it is crucial to consider the distinct roles of different SOD isoforms in colorectal cancer prognosis. According to The Human Protein Atlas, high SOD1 expression is associated with poor prognosis, SOD2 does not significantly correlate with prognosis, and low SOD3 expression is linked to worse prognosis. These isoform-specific differences should be explored in future studies to refine our understanding of antioxidant biomarkers in CRC.

Finally, the article’s comparison of results regarding antioxidant biomarkers in tissue versus serum levels is somewhat simplistic. While the study indicates that low PRX4 is associated with malignant characteristics and inflammation, it fails to explore the biological differences between tissue and serum biomarkers and their implications for result interpretation in depth. Different sample types, such as tissue and serum, may reflect distinct biological processes (17). Therefore, future research should systematically compare biomarkers from various sources and provide potential biological explanations for the observed differences, as well as discrepancies in study design. Moreover, the dual roles of PRX4 in different cancer types warrant further discussion, especially concerning heterogeneity within colorectal cancer. Given the existing literature, the role of PRX4 in various cancer types is not unidirectional (18). Consequently, the translation of PRX4 into a clinically viable predictive biomarker for colorectal cancer necessitates meticulous and systematic further investigation. Another significant concern is the use of protein expression units in the figure, which are unclear and inconsistent with the existing literature. The study fails to provide a clear rationale for the cut-off points used to categorize SOD and PRX4 expression levels. Given that protein levels were measured, the choice of units and thresholds complicates the interpretation of the results and makes it difficult to compare with established benchmarks in the field. Future research should standardize these units and carefully justify the selection of cut-off points to ensure comparability with other studies. Moreover, sensitivity analyses regarding these thresholds should be conducted to assess their impact on the study’s conclusions.

This study provides a new perspective on prognostic evaluation in colorectal cancer by establishing associations between antioxidant biomarkers, tumor aggressiveness, inflammatory responses, and clinical outcomes. These findings offer novel evidence regarding the role of antioxidant therapies and biomarkers in cancer, highlighting their potential clinical applications. If future multi-center studies with larger sample sizes can further validate these results, biomarkers such as SOD and PRX4 may emerge as predictive indicators for patients with colorectal cancer, thereby supporting the advancement of precision medicine.

To enhance the reliability and generalizability of this research, several improvements are suggested. First, expanding the sample size and conducting multi-center prospective studies are essential. The current sample size is limited to 70 cases, which restricts the applicability of the study’s conclusions. Future research should aim for a minimum sample size of at least 200 cases to increase statistical power and external validity. Second, the introduction of a dynamic monitoring design is recommended, where measurements of antioxidant biomarkers are taken at multiple time points—before treatment, after treatment, and during follow-up. This approach will help elucidate the temporal effects of antioxidant biomarkers on the progression of colorectal cancer and treatment response. Additionally, the study currently employs the first quartile as a cutoff for analysis, but this method may lack stability in smaller samples. Future research should consider conducting cutoff sensitivity analyses and exploring data-driven optimal split points to enhance the robustness and reliability of the findings. Finally, future studies should incorporate the distinct roles of different SOD isoforms (SOD1, SOD2, and SOD3) in colorectal cancer prognosis, as their specific contributions may influence biomarker development and clinical applications.

## 3 Conclusion

In summary, this study makes an innovative contribution to exploring the potential prognostic value of antioxidant biomarkers in colorectal cancer. However, limitations in sample size, methodological design, and statistical analysis may hinder the robustness and broad applicability of the conclusions. Future studies should expand sample sizes, employ dynamic monitoring across multiple time points, and conduct multi-center validations to further investigate the clinical application potential of biomarkers such as SOD and PRX4. Furthermore, enhancing mechanistic research and integrating a multi-dimensional antioxidant network will contribute to a more comprehensive assessment of the roles of antioxidants in colorectal cancer.
